# Association between periodontitis stages and self-reported diseases in a Norwegian population: the HUNT study

**DOI:** 10.1186/s12903-023-03743-z

**Published:** 2023-12-13

**Authors:** Ida Haukåen Stødle, Abhijit Sen, Hedda Høvik, Anders Verket, Odd Carsten Koldsland

**Affiliations:** 1 Department of Periodontology, Institute of Clinical Dentistry, Faculty of Dentistry, University of Oslo, Geitmyrsveien 69, 0455 Oslo, Norway; 2 Center for Oral Health Services and Research, Mid-Norway (TkMidt), Trondheim, Norway

**Keywords:** Periodontitis, Stage, Non-communicable diseases, Association, Odds ratio, HUNT4

## Abstract

**Background:**

The relationships between periodontitis and non-communicable diseases (NCDs) have been investigated through several different case-definitions. The differences in methodology may have hindered the basis of comparison between these studies. The classification from the 2017 World Workshop on the Classification of Periodontal and Peri-implant Diseases and Conditions offers a unison platform that may facilitate future comparison of such research. The present study aimed to reproduce associations between periodontitis and other NCDs using the 2017 Classification, in the Trøndelag Health Study (HUNT).

**Material and methods:**

The fourth HUNT-survey was carried out between 2017 and 2019. Clinical variables, blood samples and answers to questionnaires were collected from 4933 participants. Periodontal status was assessed based on the latest staging system, and its associations with NCDs were estimated by logistic regression models adjusted for potential confounders.

**Results:**

Compared to no or Stage I periodontitis, participants with Stage III/IV periodontitis (radiographic bone loss exceeding 33%) were associated with cardiovascular disease, hyperglycemia in participants with diabetes and chronic obstructive pulmonary disease (COPD)/emphysema. Associations with hyperglycemia in participants with diabetes and COPD/emphysema were also observed in participants with Stage II periodontitis. The only observed association when considering never-smokers alone, was with COPD/emphysema.

**Conclusion:**

Periodontitis Stage II and III/IV were associated with major NCDs. Effect sizes increased with increasing periodontitis stages, which implies greater occurrence of coincident comorbidities in patients with severe periodontitis.

**Supplementary Information:**

The online version contains supplementary material available at 10.1186/s12903-023-03743-z.

## Introduction

Periodontitis is a destructive inflammatory disease, causing loss of supporting bone and soft tissue surrounding the teeth, and may lead to tooth loss if left untreated. Periodontitis has been reported in 40% of the US adult population [1], and severe periodontitis in 7.4% worldwide [2]. In a recent cross-sectional study based on the 2017 Classification, Stage III and Stage IV periodontitis combined was observed in 17% of adults in a Norwegian population [3].

Periodontitis is associated with several non-communicable diseases (NCDs) [4–6]. Shared immunological and inflammatory reactions in the host or bacteremia caused by pathogens of periodontal origin are suggested mechanisms of these associations. Disease activity is modulated through production of inflammatory factors, including interleukins, prostaglandins, and matrix metalloproteinases [7].

The supporting evidence of an association between periodontitis and systemic diseases includes several mechanisms. It is suggested that elevated systemic inflammation, observed through acute-phase proteins and oxidative stress biomarkers is a result of organisms entering the circulation. AGE-RAGE interactions are central in the plausible mechanistic links between periodontitis and diabetes, and leads to the exaggerated inflammatory response and periodontal tissue destruction seen in diabetics. Moreover, diabetes is associated with elevated levels of several cytokines and other mediators in saliva and gingival crevicular fluid (GCF) [4]. Translocated circulating oral microbiota is also thought to impact on development of atherothrombogenesis through induction of systemic inflammation [5]. *P. gingivalis* has been shown to accelerate atherosclerosis in animal models, and to induce aorta fatty streaks after bacteremia [8, 9]. Further mechanistic evidence includes elevated levels of antibodies that have been shown to cross-react with antigens in cardiovascular tissues and increased production of reactive oxygen species (ROS) in peripheral neutrophils in periodontitis patients [8, 10].

The 2017 Classification redefines the outline of case criteria and disease severities. The aim of this cross-sectional study was to reproduce associations between periodontitis with the use of the 2017 Classification and several prevalent NCDs.

## Material and methods

### Study design and population

**Fig. 1:**
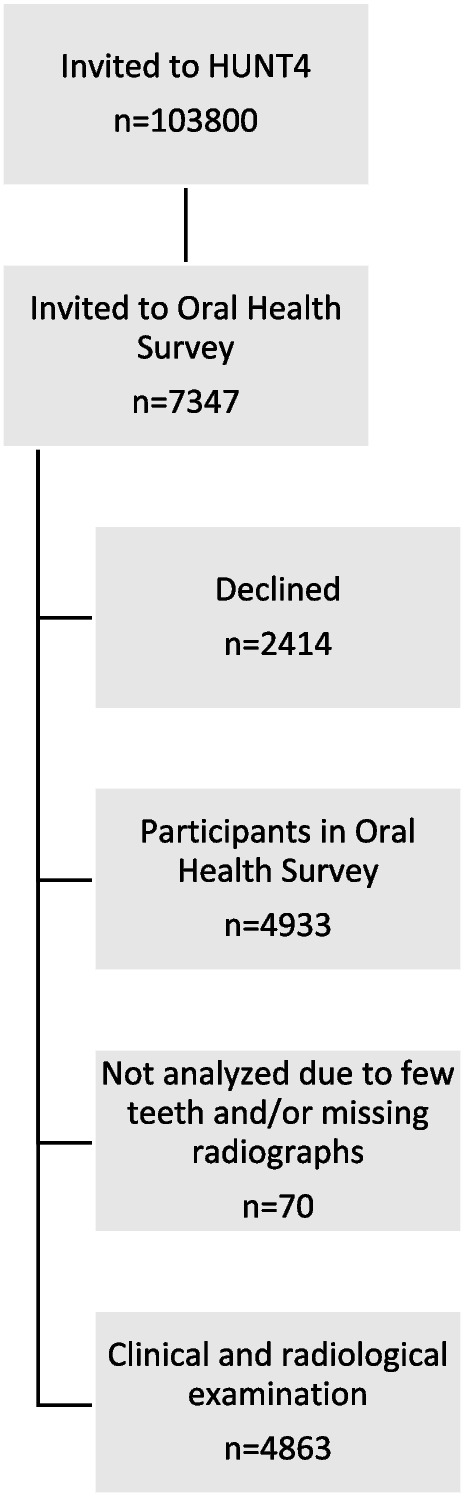
Study participants

The investigated population was a part of The Trøndelag Health Study (HUNT). HUNT is a longitudinal population-based study which constitutes a large database of questionnaire data, clinical measurements and biological samples [11–13]. The present cross-sectional study was based on data from HUNT4 which was conducted between September, 2017 and February, 2019. The county of Nord-Trøndelag had 137,233 residents in 2017. All residents in the county, turning 20 years within the year of participation and older (*n* = 103,800), were invited of whom 56,042 (54.0%) participated [13]. At HUNT4 field stations in six larger municipalities (Stjørdal, Levanger, Verdal, Steinkjer, Nærøy, and Namsos) a random sample were invited to the oral health examination, HUNT4 Oral health study, where periodontal examination was included for the first time. This constituted a subpopulation of 7347 HUNT4 participants. Finally, 4933 (67.1%) participated in clinical and/or radiographic oral examinations (Fig. 1).

### Periodontal examination, questionnaires and blood samples

Periodontal and systemic health status was assessed by clinical and radiological examination, answers to questionnaires and by collection of blood samples. Trained, calibrated dentists/dental hygienists (*n* = 19) performed all clinical examinations, as described in a previous publication [3]. Briefly, periodontal probing depths (PPD), bleeding on probing (BoP) and suppuration were registered at six surfaces per tooth in addition to mobility grade 2 and 3 [14, 15]. Third molars and root remnants were excluded. PPD was measured with a WHO periodontal probe (LM 550BSI Probe WHO ErgoNorm, LM-Instruments, Parainen, Finland), and was recorded in the following intervals: 0–3.5 mm, > 3.5–5.5 mm, > 5.5–8.5 mm, > 8.5–11.5 mm, > 11.5 mm. Calibration of the clinical examiners consisted of theoretical lectures and repeated clinical practical training supervised by experienced, certified periodontists (OCK, AV). The radiographic examination consisted of four bitewings (BW) and one orthopantomogram (OPG) radiograph. The OPGs were obtained with a panoramic imaging unit, Planmeca ProOne (Planmeca Oy, Helsinki, Finland). The BW radiographs were obtained using an intraoral imaging unit, Planmeca Intra (Planmeca Oy, Helsinki, Finland), with a rectangular collimator (length 35 cm) and an intraoral sensor, ProSensor HD (Planmeca Oy, Helsinki, Finland). Examination of radiographs was performed by three calibrated periodontists (IHS, AV and OCK). Inter-investigator calibration was performed on radiographs and clinical variables from 70 participants. Each investigator registered percentage of bone loss for every tooth in the dentition and determined stage. In cases of disagreement, consensus was reached by discussions in plenary [3].

### Periodontitis definition and classification of stages

A description of case definition and periodontitis classification has been published previously [3], in short; A periodontitis case was defined as a subject with a distance between the cementum enamel junction (CEJ) and the alveolar bone crest (AC) exceeding 1.5 mm at ≥ 2 non-adjacent teeth. This was determined from BW radiographs and was considered “detectable interproximal bone loss”. The distance from the CEJ to the top of the AC where the periodontal ligament (PDL) presented normal width [16], and the distance from the CEJ to the radiographic apex were measured in mm in the entire dentition to calculate the percentage of bone loss for all teeth. The radiographic bone loss (RBL) for each tooth was recorded in the following intervals: < 15%, 15–33%, > 33%. The most severely affected tooth was used to determine initial stage. If BW examination was not performed, the distance between the CEJ and the AC was determined from OPGs (*n* = 64). For participants with only BW radiographic examination available (*n* = 22), the percentage of bone loss was determined according to a study of average root lengths [17]. All participants were classified based on the 2017 World Workshop on the Classification of Periodontal and Peri-implant Diseases and Conditions [18–20]. Determination of final stage was based on RBL in OPGs, the number of teeth considered lost due to periodontitis and relevant complexity factors. The complexity factors included vertical bone loss (radiographic defects ≥ 3 mm deep and ≤ 3 mm wide), furcation grade ≥ 2 (overt radiolucency evident in the furcation areas) [21, 22], bite collapse/drifting/flaring (≥ 3 teeth with obvious change of position and/or mobility grade ≥ 2 within the same sextant, in combination with presence of periodontal bone loss likely to cause this condition) and PPD > 5.5 mm (used to identify Stage III when the RBL corresponded to Stage II definition) [3]. The complexity factors vertical bone loss, furcation involvement and bite collapse/drifting/flaring were determined from OPG radiographs. If clinical parameters were not available, determination of stage was based on radiographic evaluation only (*n* = 71).

### Outcomes

Self-reported history of NCDs (CVD, rheumatoid disorder, COPD/emphysema) and hyperglycemia in self-reported diabetics, were the study endpoints. CVD was defined as a composite of myocardial infarction and/or angina pectoris and/or apoplexia [23]. Rheumatoid disorder was defined as a composite of rheumatoid arthritis and/or ankylosing spondylitis. Hyperglycemia (HbA1c ≥ 48 mmol/mol) was described in subjects with self-reported diabetes.

### Exposure of interest

The exposure periodontitis stage was grouped into three categories (no periodontitis/Stage I, Stage II and Stage III/IV).

### Covariates

Selected confounders included age (continuous), sex, smoking (never smokers, < 10, 10–20, > 20 pack years), years of education (9–10 years, 11–13 years, college/university), total gross household income (< 40,000, 40–70000, > 70,000 EUR), BMI (kg/m^2^, continuous), hypertension (> 140/90 mmHg) and HbA1c-level (continuous) [24–27]. Information about sociodemographic and lifestyle factors were assessed via self-reported questionnaires. Anthropometric variables including weight, height and blood pressure were measured by trained personnel at HUNT4 field stations. Blood pressure measurements were based on automatic oscillometric method, using Dinamap CARESCAPE V100 (GE Healthcare, Chicago, US) with GE TruSignal for pulse oximetry. Blood pressure was measured in a sitting position according to standardized methods. Three consecutive automatic oscillometric BP-measurements were recorded at 1-min intervals and the mean of the second and the third readings were calculated. Blood pressure was registered to the nearest 2 mmHg. Blood samples were drawn and analyzed for HbA1c in non-fasting serum or whole blood. Two separate enzymatic analysis; HbA1c and THb, were used to calculate the percentage (NGSP-units) and the hemoglobinfraction (IFCC-units) of HbA1c (Reagent kit; 4P52-21 Hemoglobin A1c, Multigent, Abbot Laboratories, USA). Missing responses in the questionnaires were reported as “Missing information”. All variables are listed in Table 1.

**Table 1:** Characteristics of study population, by periodontal disease

Characteristics	Total (*n* = 4933)^a^	No or Stage I periodontitis (*n* = 1969)	Stage II periodontitis (*n* = 2028)	Stage III/IV periodontitis (*n* = 866)	p-value
Age (years)	51.8 (16.6)	38.2 (12.3)	58.5 (11.9)	65.9 (10.8)	< 0.001
Sex					
Male	44.1 (42.7–45.4)	43.8 (41.6–46.1)	43.9 (41.8–46.1)	46.0 (42.6–49.3)	0.12
Female	55.9 (54.5–57.3)	56.2 (53.9–58.4)	56.1 (53.9–58.2)	54.0 (50.7–57.4)	
Education					
Primary (9–10 years)	7.3 (6.6–8.1)	2.8 (2.6–3.7)	7.7 (6.6–8.9)	14.8 (12.5–17.3)	< 0.001
Secondary (11–13 years)	47.2 (45.8–48.6)	45.1 (42.9–47.3)	47.8 (45.6–50.0)	50.7 (47.3–54.1)	
University/College	45.0 (43.6–46.4)	51.6 (49.3–53.8)	44.1 (41.9–46.3)	33.7 (30.6–37.0)	
Missing information	0.5 (0.3–0.8)	0.5 (0.2–0.9)	0.4 (0.2–0.8)	0.8 (0.3–1.7)	
Income, total household					
< 40.000 EUR	25.7 (24.5–26.9)	23.0 (21.1–24.9)	22.7 (20.9–24.6)	37.3 (34.1–40.6)	< 0.001
40.000–70.000 EUR	28.4 (27.1–29.7)	24.1 (22.2–26.1)	29.7 (27.7–31.7)	35.8 (32.6–39.1)	
> 70.000 EUR	43.8 (42.4–45.1)	50.9 (48.7–53.2)	45.7 (43.5–47.9)	24.1 (21.3–27.1)	
Missing information	2.1 (1.8–2.6)	2.0 (1.4–2.7)	1.9 (1.4–2.6)	2.8 (1.8–4.1)	
BMI (kg/m^2^)	27.1 (4.6)	26.6 (4.9)	27.2 (4.3)	27.6 (4.6)	< 0.001
Missing information	0.3 (0.2–0.5)	0.2 (0.03–0.4)	0.3 (0.1–0.6)	0.3 (0.1–1.0)	
Smoking					
Never smokers	45.3 (43.9–46.7)	55.9 (53.6–58.1)	43.3 (41.2–45.5)	26.4 (23.5–29.5)	< 0.001
Pack years					
> 0.0–9.9	24.6 (23.4–25.8)	20.4 (18.7–22.3)	29.0 (27.0–31.0)	24.4 (21.5–27.4)	
10–20	10.9 (10.0–11.8)	4.4 (3.5–5.4)	13.0 (11.6–14.6)	20.6 (17.9–23.4)	
> 20	7.7 (7.0–8.5)	1.1 (0.7–1.6)	6.9 (5.8–8.1)	23.6 (20.8–26.5)	
Missing information	11.6 (10.7–12.5)	18.3 (16.6–20.1)	7.7 (6.6–9.0)	5.1 (3.7–6.8)	
HbA1c (mmol/mol)	34.1 (6.0)	32.0 (4.4)	35.0 (6.1)	36.5 (7.0)	< 0.001
HbA1c (%)	5.3 (0.5)	5.1 (0.4)	5.3 (0.6)	5.5 (0.6)	
Missing information	1.0 (0.8–1.4)	0.9 (0.5–1.4)	1.2 (0.8–1.8)	1.0 (0.5–2.0)	
Hypertension	20.7 (19.6–21.9)	9.3 (8.1–10.7)	25.5 (23.6–27.4)	35.1 (31.9–38.4)	< 0.001
Missing information	0.2 (0.1–0.4)	0.1 (0.01–0.4)	0.2 (0.1–0.5)	0.5 (0.1–1.2)	
Myocardial infarction/apoplexia/angina	6.5 (5.8–7.2)	1.6 (1.1–2.3)	7.5 (6.4–8.7)	14.0 (11.7–16.5)	< 0.001
Missing information	2.8 (2.4–3.3)	1.6 (1.1–2.2)	3.2 (2.5–4.1)	4.3 (3.0–5.8)	
HbA1c ≥ 48 mmol/mol (6.5%) in diabetics	2.7 (2.2–3.2)	1.7 (1.2–2.4)	5.5 (4.6–6.6)	8.2 (6.5–10.2)	< 0.001
Missing information	2.3 (1.9–2.8)	1.1 (0.7–1.6)	1.2 (0.8–1.8)	2.1 (1.2–3.3)	
Rheumatoid disorder	5.9 (5.3–6.6)	3.0 (2.3–3.8)	7.1 (6.0–8.3)	9.2 (7.4–11.4)	< 0.001
Missing information	3.4 (2.9–3.9)	1.9 (1.4–2.6)	3.8 (3.0–4.7)	5.0 (3.6–6.6)	
COPD/emphysema	2.1 (1.7–2.5)	0.2 (0.03–0.4)	2.1 (1.2–2.8)	5.9 (4.4–7.7)	< 0.001
Missing information	3.8 (3.3–4.4)	2.2 (1.6–2.9)	4.3 (3.5–5.3)	5.7 (4.2–7.4)	
Number of teeth	25.4 (4.3)	27.1 (2.1)	25.5 (3.4)	22.3 (5.3)	< 0.001
Number of participants with					
PPD ≥ 4 mm in ≥ 1 site	48.6 (47.2–50.0)	32.8 (30.7–34.9)	51.1 (48.9–53.3)	81.2 (78.4–83.7)	< 0.001
PPD ≥ 6 mm in ≥ 1 site	9.4 (8.6–10.3)	1.7 (1.2–2.4)	3.5 (2.7–4.3)	41.6 (38.3–44.9)	
Missing information	2.3 (1.9–2.7)	1.2 (0.8–1.8)	1.5 (1.0–2.1)	2.0 (1.1–3.1)	

Data are presented as mean (SD) or percentage (95% CI). Differences among groups were analyzed by One-way ANOVA for continuous variables and by × 2 for categorical variables

*Abbreviations*: *SD* standard deviation, *CI* confidence interval, *PPD* periodontal probing depth

^a^70 participants (1.4%) were considered ineligible for periodontal diagnosis, i.e., edentulous participants or participants missing radiographs

### Statistical analyses

Descriptive statistics are presented as mean and standard deviation for continuous variables, and percentages with 95% confidence intervals for categorical variables. The one-way ANOVA test was used to assess significant differences across group means for continuous variables and Pearson chi-square test was used for categorical variables. Logistic regression analyses were used to assess associations between periodontitis stages and CVD (0: no, 1: history of CVD), hyperglycemia in diabetics (0: no, 1: HbA1c ≥ 48 mmol/mol (6.5%) in self-reported diabetics), rheumatoid disorders (0: no, 1: history of rheumatoid disorders) and COPD/emphysema (0: no, 1: history of COPD/emphysema). All models were adjusted for potential confounders, listed in footnotes of Table 2. Selection of confounders was based on variables known to be associated with periodontitis or any of the outcomes. For testing p for trend, periodontitis stages were included as a continuous variable. Sensitivity analyses were conducted to assess the robustness of the findings by i) exclusion of participants 75 years and older, ii) separate analysis of non-diabetics, iii) separate analysis of participants reporting diabetes diagnosis, but without considering HbA1c-level and iv) separate analysis of never-smokers. The odds ratios (OR) with corresponding 95% confidence intervals were computed.

**Table 2:** Association between periodontitis stages and cardiovascular disease, diabetes, rheumatoid disorders and COPD/emphysema, by logistic regression analysis

NCD ^a,b,c^	No. of observations	Crude OR (95% CI)	No. of observations	Adjusted OR (95% CI)
Cardiovascular disease^a^				
Stage II	*n* = 4730	4.99 (4.40–7.36)	*n* = 4055	1.39 (0.88–2.22)
Stage III/IV		10.18 (6.83–15.17)		1.73 (1.04–2.89)
Per unit increase		2.84 (2.41–3.35)		1.29 (1.02–1.63)
p-linear trend		< 0.001		0.032
Diabetes, HbA1c ≥ 48 mmol/mol (6.5%) in self-reported diabetics^b^				
Stage II	*n* = 4750	4.64 (2.60–8.30)	*n* = 4114	2.12 (1.05–4.30)
Stage III/IV		8.12 (4.45–14.84)		2.56 (1.17–5.61)
Per unit increase		2.52 (1.97–3.23)		1.44 (1.03–2.03)
p-linear trend		< 0.001		0.035
Rheumatoid disorders^c^				
Stage II	*n* = 4705	2.53 (1.85–3.45)	*n* = 4083	1.20 (0.79–1.81)
Stage III/IV		3.42 (2.41–4.83)		1.08 (0.66–1.77)
Per unit increase		1.82 (1.55–2.14)		1.01 (0.80–1.28)
p-linear trend		< 0.001		0.948
COPD/emphysema^c^				
Stage II	*n* = 4130	14.52 (4.50–46.89)	*n* = 4061	4.17 (1.20–14.57)
Stage III/IV		42.68 (13.28–137.15)		5.40 (1.48–19.78)
Per unit increase		4.22 (3.08–5.78)		1.62 (1.08–2.43)
p-linear trend		< 0.001		0.020

Reference: No periodontitis/ periodontitis Stage I

*Abbreviations*: *NCD* non-communicable disease, *OR* odds ratio, *CI* confidence interval

^a^Adjusted for HbA1c-level, BMI, hypertension, age, sex, smoking (pack years), income and years of education

^b^Adjusted for BMI, hypertension, age, sex, smoking (pack years), income and years of education

^c^Adjusted for hypertension, age, sex, smoking (pack years), income and years of education

Inter-rater reliability for clinical measurement (PPD) was calculated using intraclass correlation coefficient (ICC) two-way mixed effect model, assessing absolute agreement.

ICC (95% CI) for the clinical examiners were:

0.57 (0.52–0.60), 0.68 (0.65–0.71), 0.71 (0.68–0.74), 0.77 (0.75–0.79), 0.77 (0.74–0.79), and 0.79 (0.75–0.82). Inter-rater reliability for evaluation of generalized stage and localized stage were calculated using ICC two-way mixed effect model, assessing absolute agreement.

- Generalized stage: ICC (95% CI) = 0.92 (0.88–0.95)
- Localized stage: ICC (95% CI) = 0.94 (0.91–0.96)

Inter-rater reliability for radiographic bone loss assessment (percentage of root length) was calculated using ICC two-way mixed effects, assessing consistency.

Periodontal bone loss: ICC (95% CI) = 0.95 (0.918–0.964). All statistical analyses were based on complete cases and were performed using Stata/MP 16.0 (Stata Corp., TX, USA).

### Ethical approval

The HUNT4 Survey was approved by the Norwegian Data Protection Authority. Informed consent was obtained from all participants and/or their legal guardians. The current study was performed in accordance with relevant guidelines and regulations and was evaluated and approved by the Norwegian Regional Committees for Medical and Health Research Ethics during the conception of the study and when the data collection commenced (2016/1879/REK, 2020/10417/REK, 2021/264485/REK). The paper was prepared following the STROBE guidelines.

## Results

Characteristics of the total population by periodontitis stages are presented in Table 1. Of the 4933 participants, 56% were female. The age distribution ranged from 19 to 94 years, and the mean age was 51.8 years (standard deviation (SD) 16.6). The number of edentulous participants was 33 (0.66%), and the average number of teeth present was 25.4 (SD 4.3). PPD ≥ 4 mm in at least one site was observed in 2399 participants (48.6%). PPD ≥ 6 mm in at least one site was observed in 466 participants (9.4%). For teeth considered “missing due to periodontitis”, 247 (5.0%) had lost 1–4 teeth, whereas 106 (2.1%) had lost 5 teeth or more. Dental examinations and/or radiographic assessments were not performed in 70 of the participants included in the oral health survey, leaving a total of 4863 participants for statistical analysis (Fig. 1). Significant differences across periodontitis stages in relation to demographic-, lifestyle-, anthropometric- and clinical factors was observed (Table 1). Participants with periodontitis Stage II-IV were relatively older, smoked more frequently and had lower levels of education and income compared to participants with no or Stage I periodontitis. Further, comorbid conditions and hypertension were observed more frequently for participants with periodontitis Stage II-IV.

### Association with periodontitis stages

The associations between periodontitis stages and NCDs are presented in Table 2. In the adjusted models, periodontitis Stage III/IV was associated with increased occurrence of CVD (OR, 1.73, 95% CI 1.04–2.89), hyperglycemia in diabetics (OR 2.56, 95% CI 1.17–5.61) and COPD/emphysema (OR 5.40, 95% 1.48–19.78), compared to no/Stage I periodontitis. The effect sizes for periodontitis Stage II, were relatively lower. No association was observed between periodontitis and rheumatoid disorders. Positive linear trends were observed between stages of periodontitis and CVD, hyperglycemia in diabetics, and COPD/emphysema.

### Sensitivity analyses

The analyses of participants below 75 years and of non-diabetics, corresponded to the results of the main analyses (Supplementary Tables 1 and 2). In the sensitivity analysis of participants with diabetes regardless of glycemic control, no association was observed (Supplementary Table 3). In analysis of never-smokers, associations were observed for periodontitis Stage II (OR 10.79, 95% CI 1.04–111.90) and Stage III/IV (OR 14.58, 95% CI 1.00–212.58) with COPD/emphysema (Supplementary Table 4).

## Discussion

This cross-sectional study of 4933 adult participants assessed associations between self-reported NCDs and periodontitis stages based on the 2017 Classification. The results suggest that periodontitis is associated with CVD, hyperglycemia in participants with diabetes and with COPD/emphysema related to the severity of periodontal disease. The associations were generally stronger with increasing stage severity. No association was observed between periodontitis and rheumatoid disorders.

The observed associations between periodontitis and CVD in the present investigation are consistent with other previous studies [28, 29]. The most recent study [28] used NHANES data to investigate associations between coronary heart disease/stroke (CVD) and periodontitis by the 2017 Classification of Periodontal and Peri-Implant Diseases and Conditions. The authors assessed clinical periodontal attachment loss and reported that Stage III and IV periodontitis were associated with 3.59 times greater occurrence of CVD compared to Stage I periodontitis. This is higher, but comparable to the present study (OR 1.73 (95% CI 1.04–2.89). Further, the increasing effect sizes of periodontitis stages and CVD in the present study confirm findings by Ngamdu and coworkers with OR 2.58 (95% CI 0.97–6.89) and 3.59 (95% CI 1.12–11.54), for Stage II and Stage III/IV, respectively. Studies with different design are also supportive of these findings. A large longitudinal study of Korean individuals older than 40 years [30], showed that periodontitis assessed from medical records was associated with future cardiac events in individuals without previous cardiac disease and that this association was reduced with good oral hygiene and more frequent dental visits. Improved oral hygiene behavior was suggested as a modifier of the association between periodontitis and CVD. Another follow-up study reported adverse cardiovascular events in individuals who had been treated for severe periodontitis [31]. This association was observed in individuals older than 60 years, only.

Neither sensitivity analyses of participants below 75 years nor analyses of non-diabetics produced results that differed vastly from the main analyses in the present study. The associations between CVD and periodontitis Stage II (OR 1.65, 95% CI 0.99–2.73) and Stage III/IV (OR 1.82, 95% CI 1.03–3.21) in participants younger than 75 years, are in line with a report from the PAROKRANK case–control study [29], despite the differences in study design. In PAROKRANK the association between myocardial infarction and radiologically assessed periodontal bone loss (OR 1.28 (95% CI 1.03–1.60) was assessed in patients < 75 years only, to avoid disturbance from accumulation of concomitant disorders.

There is abundant literature on the relationship between periodontitis and diabetes. It has been stated that diabetic patients in general have higher severity of periodontal disease compared to non-diabetics**,** while other studies report comparable periodontal status in patients with controlled diabetes to that of the general population [32]. A systematic review by Graziani and coworkers [33] concluded that due to heterogeneity among publications, there is still some conflicting evidence, and stated that several studies are unable to confirm periodontitis’ impact on diabetes control, incidence and complications, or that type 2 diabetes in individuals with periodontitis is associated with higher levels of HbA1c. The present observations are in line with the aforementioned statements. Stage II and Stage III/IV periodontitis were associated with hyperglycemia in self-reported diabetics in the total population. This association was also observed with Stage III/IV in the subpopulation of participants less than 75 years. When self-reported diabetes was assessed alone without consideration of glycemic control, no association was observed.

Periodontitis was not associated with rheumatoid disorders in the present study, which is in contrast to a recent systematic review and meta-analysis [34]. Hussain and co-workers emphasized that rheumatoid arthritis did not affect periodontal attachment level, but that there is moderate evidence to suggest that individuals with periodontitis have more swollen or tender joints, report more pain on visual analogue scales and have higher erythrocyte sedimentation rates, assessed by a disease activity score tool in 28 joints (DAS28). In the present analysis of rheumatoid disorders, information about joint pain and -motion, functionality, duration of symtoms and use of medication were not assessed, hence the present model design may have inflicted the contrasting findings.

The present analysis of COPD/emphysema is supportive of a Japanese 5-year cohort study [35]. The authors reported an association between severe periodontitis and COPD. Similarly, a systematic review and meta-analysis have validated associations between periodontitis and asthma, COPD and pneumonia [36]. The biological plausibility for such associations include epithelial damage to lower respiratory tract caused by periodontal pathogens and cytokine release, and neutrophilic inflammation with subsequent proteolytic destruction of connective tissue [6, 37]. It has been suggested that smoking should be considered a modifier of associations between COPD and periodontitis as smoking plays an important role in etiology in both diseases [38]. When analyzing never-smokers in the present investigation, no association with NCDs were observed with the exception of COPD/emphysema. The wide confidence intervals in the present analysis indicates uncertainty of the relationship with COPD/emphysema. Only 2.1% (*n* = 104) of the total population reported COPD/emphysema, and 51 of these 104 individuals (49%) were classified with periodontitis Stage III/IV.

The stages of the 2017 classification reflect severity of periodontitis. Accordingly, it may be used to explore a dose-dependent relationship between periodontitis stages and associated conditions, and perhaps more easily identify if there are certain stages or individuals with increased likelihood of coincident diseases. This is of relevance for patients and dental professionals and may be important in risk factor modification as part of periodontal therapy. Conversely, individuals with systemic diseases may present more severe stages of periodontitis, and awareness of this association for patients and medical doctors is encouraged. The latest staging and grading system may facilitate research on how established and potential risk indicators or determinants of periodontitis are distributed between the different stages of disease. Nevertheless, the suitability of the new classification system in epidemiological research, and its potential benefits for patients’ periodontal health has yet to be explored. The cross-sectional design of the present study will not fully reflect disadvantages of the classification in this respect.

The investigated population is representative of smaller cities and rural areas in Norway [11, 13] and the results from the present study may only be generalized to similar populations. Previous findings from the HUNT Study have shown that non-participants had lower socioeconomic status and higher prevalence of chronic diseases and mortality than those who participated [39]. This was confirmed in the latest cohort profile [13] who reported a less healthy lifestyle and inferior self-reported health and higher proportion of cardiovascular diseases, chronic obstructive pulmonary disease, diabetes and antihypertensive medication use, in non-participants. In the present analysis, total household income had a modest effect on the observed associations. Low socioeconomic status has been presented as a risk factor for periodontal disease [40], however, the magnitude may be masked in the present investigation due to inclusion of younger, healthy individuals in a generally healthy, high-income population. There was a tendency of a protective relationship between higher income and NCDs (Supplementary Table 5).

Moreover, the magnitude of the crude ORs compared to the adjusted ORs of the statistical estimates suggests that some of the independent variables have a significant effect as determinants of the outcomes, together with periodontitis stages. This should be taken into consideration when interpreting the results.

Self-reported systemic illness is a limitation in the present investigation. HUNT cohort profiles [13] indicate that when compared to hospital journal charts and registries, the sensitivity, specificity and predictive values of the self-reported information varies across diagnoses. In-depth validity studies have been conducted. For self-reported diabetes, Midthjell and co-workers [41] found that patient administered questionnaires regarding a well-defined disease, was highly reliable for epidemiological purposes. The modest inter-examiner reliability of the clinical examiners is also a limitation. On the other hand, the clinical measurements and biological samples are considered strengths. The large sample size of nearly 5000 individuals and full mouth clinical and radiographical periodontal examination and assessment by the 2017 Classification are also strengths of this study.

## Conclusion

The present investigation demonstrates that associations between periodontitis and history of CVD, diabetes and COPD/emphysema, increases with periodontitis stage severity.

### Supplementary Information

**Additional file 1: Supplementary table 1.** Association between periodontitis stages and cardiovascular disease, diabetes, rheumatoid disorders and COPD/emphysema, in participants below 75 years.

**Additional file 2: Supplementary table 2.** Association between periodontitis stages and cardiovascular disease, rheumatoid disorders and COPD/emphysema, in non-diabetics.

**Additional file 3: Supplementary table 3.** Association between periodontitis stages and diabetes, without consideration of HbA1c-levels.

**Additional file 4: Supplementary table 4.** Association between periodontitis stages and cardiovascular disease, diabetes, rheumatoid disorders and COPD/emphysema, in never-smokers.

**Additional file 5: Supplementary table 5.** Association between periodontitis stages and NCDs, by logistic regression analysis. Adjusted by five levels of income

## Data Availability

The Trøndelag Health Study (HUNT) has invited persons aged 13-100 years to four surveys between 1984 and 2019. The data are stored in HUNT databank and biological material in HUNT biobank. HUNT Research Centre has permission from the Norwegian Data Inspectorate to store and handle these data. The key identification in the data base is the personal identification number given to all Norwegians at birth or immigration, whilst de-identified data are sent to researchers upon approval of a research protocol by the Regional Ethical Committee and HUNT Research Centre. To protect participants’ privacy, HUNT Research Centre aims to limit storage of data outside HUNT databank and cannot deposit data in open repositories. HUNT databank holds precise information on all data exported to different projects and can reproduce these on request. There are no restrictions regarding data export given approval of applications to HUNT Research Centre. Provided approval from HUNT Research center, sharing of data from the present investigation will be supported by the corresponding author upon reasonable request. For more information see: www.ntnu.edu/hunt/data. Inquiries regarding access to data is directed to: kontakt@hunt.ntnu.no. https://biobankregisteret.no/#/biobankDetails/4094, project 2020/26788.

## ABBREVIATIONS

AAP: American Academy of Periodontology
AC: Alveolar bone crest
BoP: Bleeding on probing
BW: Bitewing radiograph
CEJ: Cementum enamel junction
CI: Confidence interval
CVD: Cardiovascular diseases
COPD: Chronic obstructive pulmonary disease
EFP: European Federation of Periodontology
HUNT: The Trøndelag Health Study
IFCC: International Federation of Clinical Chemistry and Laboratory Medicine
NCD: Non-communicable disease
NGSP: National Glycohemoglobin Standardization Program
OR: Odds ratio
OPG: Orthopantomogram
PDL: Periodontal ligament
PPD.: Periodontal probing depth
RBL: Radiographic bone loss
SD: Standard deviation
WHF: World Heart Federation
