# Supplementary material for: Association between periodontitis stages and self-reported diseases in a Norwegian population: the HUNT study
Source: BMC Oral Health. 2023 Dec 13;23:999. doi: 10.1186/s12903-023-03743-z (PMC10720083; doi:10.1186/s12903-023-03743-z)
Supplement: Supplementary file 1 — Additional file 1: Supplementary table 1. Association between periodontitis stages and cardiovascular disease, diabetes, rheumatoid disorders and COPD/emphysema, in participants below 75 years. [file 12903_2023_3743_MOESM1_ESM.docx]

Supplementary table 1. Association between periodontitis stages and cardiovascular disease, diabetes, rheumatoid disorders and COPD/emphysema, in participants below 75 years

| NCD ^1,2,3^ | No. of observations | Crude OR (95% CI) | No. of observations | Adjusted OR (95% CI) |
| --- | --- | --- | --- | --- |
| Cardiovascular disease^1^  Stage II  Stage III/IV | n=4420 | 4.98 (3.28-7.54)  8.49 (5.45-13.23) | n=3782 | 1.65 (0.99-2.73)  1.82 (1.03-3.21) |
| Diabetes, HbA1c≥48 mmol/mol (6.5%) in self-reported diabetics^2^  Stage II  Stage III/IV | n=4429 | 4.31 (2.39-7.76)  7.85 (4.22-14.62) | n=3829 | 1.87 (0.91-3.86)  2.31 (1.03-5.18) |
| Rheumatoid disorders^3^  Stage II  Stage III/IV | n=4400 | 2.37 (1.73-3.25)  3.16 (2.18-4.57) | n=3812 | 1.12 (0.74-1.72)  1.07 (0.64-1.80) |
| COPD/emphysema^3^  Stage II  Stage III/IV | n=4382 | 13.79 (1.28-82.24)  37.49 (11.52-122.01) | n=3795 | 4.09 (1.15-14.55)  4.75 (1.26-17.92) |

Note: Reference: No periodontitis/ periodontitis Stage I

^1^ Adjusted for HbA1c-level, BMI, hypertension, age, sex, smoking (pack years), income and years of education

^2^ Adjusted for BMI, hypertension, age, sex, smoking (pack years), income and years of education

^3^ Adjusted for hypertension, age, sex, smoking (pack years), income and years of education

Abbreviations: NCD, non-communicable disease; OR, odds ratio; CI, confidence interval
