# Supplementary material for: Association between periodontitis stages and self-reported diseases in a Norwegian population: the HUNT study
Source: BMC Oral Health. 2023 Dec 13;23:999. doi: 10.1186/s12903-023-03743-z (PMC10720083; doi:10.1186/s12903-023-03743-z)
Supplement: Supplementary file 2 — Additional file 2: Supplementary table 2. Association between periodontitis stages and cardiovascular disease, rheumatoid disorders and COPD/emphysema, in non-diabetics. [file 12903_2023_3743_MOESM2_ESM.docx]

Supplementary table 2. Association between periodontitis stages and cardiovascular disease, rheumatoid disorders and COPD/emphysema, in non-diabetics

| NCD ^1,2,3^ | No. of observations | Crude OR (95% CI) | No. of observations | Adjusted OR (95% CI) |
| --- | --- | --- | --- | --- |
| Cardiovascular disease^1^  Stage II  Stage III/IV | n=4525 | 4.69 (3.16-6.97)  9.56 (6.34-14.42) | n=3871 | 1.32 (0.82-2.12)  1.75 (1.03-2.98) |
| Rheumatoid disorders^3^  Stage II  Stage III/IV | n=4503 | 2.59 (1.88-3.56)  3.46 (2.41-4.96) | n=3899 | 1.21 (0.79-1.85)  1.06 (0.63-1.77) |
| COPD/emphysema^3^  Stage II  Stage III/IV | n=4483 | 12.22 (3.75-39.79)  38.19 (11.81-123.48) | n=3879 | 3.77 (1.07-13.32)  4.91 (1.32-18.31) |

Note: Reference: No periodontitis/ periodontitis Stage I

^1^ Adjusted for HbA1c-level, BMI, hypertension, age, sex, smoking (pack years), income and years of education

^3^ Adjusted for hypertension, age, sex, smoking (pack years), income and years of education

Abbreviations: NCD, non-communicable disease; OR, odds ratio; CI, confidence interval
