# Supplementary material for: Association between periodontitis stages and self-reported diseases in a Norwegian population: the HUNT study
Source: BMC Oral Health. 2023 Dec 13;23:999. doi: 10.1186/s12903-023-03743-z (PMC10720083; doi:10.1186/s12903-023-03743-z)
Supplement: Supplementary file 3 — Additional file 3: Supplementary table 3. Association between periodontitis stages and diabetes, without consideration of HbA1c-levels. [file 12903_2023_3743_MOESM3_ESM.docx]

Supplementary table 3. Association between periodontitis stages and diabetes, without consideration of HbA1c-levels

| NCD ^1,2,3^ | No. of observations | Crude OR (95% CI) | No. of observations | Adjusted OR (95% CI) |
| --- | --- | --- | --- | --- |
| Diabetes^2^  Stage II  Stage III/IV | n=4800 | 3.33 (2.26-4.92)  5.14 (3.39-7.81) | n=4155 | 1.53 (0.94-2.51)  1.59 (0.90-2.80) |

Note: Reference: No periodontitis/ periodontitis Stage I

^2^ Adjusted for BMI, hypertension, age, sex, smoking (pack years), income and years of education

Abbreviations: NCD, non-communicable disease; OR, odds ratio; CI, confidence interval
