# Supplementary material for: Association between periodontitis stages and self-reported diseases in a Norwegian population: the HUNT study
Source: BMC Oral Health. 2023 Dec 13;23:999. doi: 10.1186/s12903-023-03743-z (PMC10720083; doi:10.1186/s12903-023-03743-z)
Supplement: Supplementary file 4 — Additional file 4: Supplementary table 4. Association between periodontitis stages and cardiovascular disease, diabetes, rheumatoid disorders and COPD/emphysema, in never-smokers. [file 12903_2023_3743_MOESM4_ESM.docx]

Supplementary table 4. Association between periodontitis stages and cardiovascular disease, diabetes, rheumatoid disorders and COPD/emphysema, in never-smokers

| NCD ^1,2,3^ | No. of observations | Crude OR (95% CI) | No. of observations | Adjusted OR (95% CI) |
| --- | --- | --- | --- | --- |
| Cardiovascular disease^1^  Stage II  Stage III/IV | n=2156 | 3.57 (2.09-6.10)  8.13 (4.45-14.85) | n=2088 | 0.69 (0.36-1.33)  0.94 (0.43-2.03) |
| Diabetes, HbA1c≥48 mmol/mol (6.5%) in self-reported diabetics^2^  Stage II  Stage III/IV | n=2157 | 2.54 (1.08-5.96)  7.72 (3.12-19.11) | n=2109 | 1.30 (0.45-3.72)  3.17 (0.97-10.43) |
| Rheumatoid disorders^3^  Stage II  Stage III/IV | n=2150 | 2.35 (1.43-3.87)  3.08 (1.60-5.95) | n=2108 | 0.79 (0.42-1.48)  0.68 (0.30-1.56) |
| COPD/emphysema^3^  Stage II  Stage III/IV | n=2139 | 10.26 (1.28-82.24)  14.92 (1.54-144.08) | n=2097 | 10.79 (1.04-111.90)  14.58 (1.00-212.58) |

Note: Reference: No periodontitis/ periodontitis Stage I

^1^ Adjusted for HbA1c-level, BMI, hypertension, age, sex, smoking (pack years), income and years of education

^2^ Adjusted for BMI, hypertension, age, sex, smoking (pack years), income and years of education

^3^ Adjusted for hypertension, age, sex, smoking (pack years), income and years of education

Abbreviations: NCD, non-communicable disease; OR, odds ratio; CI, confidence interval
