# Supplementary material for: Association between periodontitis stages and self-reported diseases in a Norwegian population: the HUNT study
Source: BMC Oral Health. 2023 Dec 13;23:999. doi: 10.1186/s12903-023-03743-z (PMC10720083; doi:10.1186/s12903-023-03743-z)
Supplement: Supplementary file 5 — Additional file 5: Supplementary table 5. Association between periodontitis stages and NCDs, by logistic regression analysis. Adjusted by five levels of income [file 12903_2023_3743_MOESM5_ESM.docx]

Supplementary table 5. Association between periodontitis stages and NCDs, by logistic regression analysis

|  |  | CVD |  | Diabetes |  | Rheumatoid disorders |  | COPD/emphysema |  |
| --- | --- | --- | --- | --- | --- | --- | --- | --- | --- |
|  |  | Adjusted OR (95% CI) |  | Adjusted OR (95% CI) |  | Adjusted OR (95% CI) |  | Adjusted OR (95% CI) |  |
| Periodontitis  Stage 0/I  Stage II  Stage III/IV  Sex  Women  Men  Age, by one year increase  BMI, by one unit (kg/m)^2^ increase  Hypertension, >140/90 mmHg  No hypertension  Hypertension  HbA1c, by one mmol/mol increase  Smoking, pack years  Never smokers  <10  10-20  >20  Education  9-10 years  11-13 years  College  Income, tot. household, Euro  ≤20000  21000-39000  40000-70000  71000-90000  ≥90000 |  | 1.00 (Reference)  1.41 (0.88-2.25)  1.73 (1.03-2.89)  1.00 (Reference)  2.48 (1.87-3.30)  1.06 (1.04-1.07)  1.05 (1.01-1.08)  1.00 (Reference)  0.80 (0.59-1.07)  1.03 (1.01-1.04)  1.00 (Reference)  1.20 (0.86-1.67)  1.13 (0.76-1.70)  1.84 (1.24-2.72)  1.00 (Reference)  0.82 (0.53-1.27)  1.17 (0.73-1.86)  1.00 (Reference)  0.74 (0.42-1.28)  0.72 (0.41-1.26)  0.75 (0.41-1.38)  0.43 (0.22-0.86) |  | 1.00 (Reference)  2.19 (1.08-4.45)  2.67 (1.21-5.87)  1.00 (Reference)  1.96 (1.31-2.94)  1.02 (1.001-1.04)  1.12 (1.07-1.16)  1.00 (Reference)  1.37 (0.91-2.06)  1.00 (Reference)  1.35 (0.82-2.22)  1.67 (0.95-2.92)  1.50 (0.82-2.75)  1.00 (Reference)  0.69 (0.40-1.20)  0.58 (0.30-1.09)  1.00 (Reference)  0.58 (0.30-1.11)  0.39 (0.20-0.75)  0.29 (0.13-0.65)  0.35 (0.15-0.82) |  | 1.00 (Reference)  1.21 (0.80-1.83)  1.08 (0.66-1.77)  1.00 (Reference)  0.65 (0.49-0.85)  1.03 (1.02-1.05)  1.00 (Reference)  1.12 (0.83-1.52)  1.00 (Reference)  1.76 (1.29-2.42)  1.56 (1.04-2.34)  1.80 (1.15-2.82)  1.00 (Reference)  1.22 (0.76-1.98)  1.15 (0.69-1.92)  1.00 (Reference)  0.80 (0.46-1.37)  0.78 (0.46-1.34)  0.63 (0.35-1.14)  0.49 (0.26-0.93) |  | 1.00 (Reference)  4.20 (1.20-14.66)  5.44 (1.48-19.92)  1.00 (Reference)  1.35 (0.86-2.13)  1.03 (1.004-1.05)  1.00 (Reference)  0.89 (0.54-1.44)  1.00 (Reference)  1.72 (0.80-3.72)  4.55 (2.20-9.40)  9.02 (4.49-18.15)  1.00 (Reference)  0.61 (0.34-1.09)  0.48 (0.24-0.97)  1.00 (Reference)  0.88 (0.38-2.06)  0.77 (0.33-1.79)  0.41 (0.14-1.20)  0.42 (0.13-1.36) |  |

Note: The analysis is adjusted by five levels of income

Abbreviations: NCD, non-communicable disease; OR, odds ratio; CI, confidence interval
